# Cost-effectiveness of MR-mammography as a solitary imaging technique in women with dense breasts: an economic evaluation of the prospective TK-Study

**DOI:** 10.1007/s00330-020-07129-5

**Published:** 2020-08-28

**Authors:** Matthias F. Froelich, Clemens G. Kaiser

**Affiliations:** Institute of Clinical Radiology and Nuclear Medicine, University Medical Centre Mannheim, Medical Faculty Mannheim - University of Heidelberg, Theodor-Kutzer-Ufer 1-3, 68167 Mannheim, Germany

**Keywords:** Costs and cost analysis, Economics, Magnetic resonance imaging, Breast neoplasms, Cost-benefit analysis

## Abstract

**Objectives:**

To evaluate the economic implications of our previous study on the use of MR-mammography (MRM) as a solitary imaging tool in women at intermediate risk due to dense breasts.

**Background:**

In our previous study, we found MRM to be a specific diagnostic tool with high accuracy in patients with dense breasts representing a patient collective at intermediate risk of breast cancer. For this study, we examined whether MRM is an economical alternative.

**Methods:**

For the determination of outcomes and costs, a decision model based on potential diagnostic results of MRM was developed. Quality of life was estimated in a Markov chain model distinguishing between the absence of malignancy, the presence of malignancy, and death. Input parameters were utilized from the prospective TK-Study. To investigate the economic impact of MRM, overall costs in € and outcomes of MRM in quality-adjusted life years (QALYs) were estimated. A deterministic sensitivity analysis was performed.

**Results:**

MRM was associated with expected costs of 1650.48 € in the 5-year period and an expected cumulative outcome of 4.69 QALYs. A true positive diagnosis resulted in significantly lower costs and a higher quality of life when compared to the consequences of a false negative result. In the deterministic sensitivity analysis, treatment costs had more impact on overall costs than the costs of MRM. The total costs per patient remained below 2500 € in the 5-year period.

**Conclusion:**

MRM, as a solitary imaging tool in patients at intermediate risk due to dense breasts, is economically feasible.

**Key Points:**

• *In patients with dense breasts (i.e., patients at intermediate risk of breast cancer), the relative cost of MR-mammography examinations only had moderate impact on overall costs.*

• *This is due to cost-savings through the application of a sensitive imaging technique resulting in an optimized staging and therapy planning.*

• *MR-mammography, unaccompanied by mammography or ultrasound in patients with dense breasts, was economically feasible in our analysis.*

## Introduction

Screening women for breast cancer has been a classical domain of conventional imaging. X-ray-based mammography (XM) herein represents the main diagnostic pillar as it is considered affordable, accompanied by a reasonable sensitivity and specificity level in order to cope with the number of patients at aim [1–3].

Recently, cost-effectiveness analyses of XM for breast cancer screening have gained further interest [4–6].

MR-mammography (MRM) today is accepted to be a highly accurate imaging technique in the detection of breast cancer [7–11].

However, it has so far not been recommended by the major breast societies as a standard technique for any other indication than patients at high risk of developing breast cancer and as an occasional problem solver [12, 13].

Reasons may have been some studies suggesting MRM to be generally unspecific and therefore not cost-effective, considering the higher “per examination” cost [14].

However, some publications in recent years indicated a possible new role for MRM in patients with BI-RADS 3 or 4 findings as a problem solver [15] or in patients at intermediate risk due to dense breast tissue [9, 16].

To our knowledge, the first study examining conventionally difficultly assessable patients at intermediate risk due to dense breasts with MRM, unaccompanied by conventional imaging as a solitary imaging tool, was the TK-Study [9].

The study was able to demonstrate sensitivity levels for MRM of 100%, as well as specificity levels of 97% in approximately 1200 patients, either biopsied or followed-up as the gold standard of reference.

In line with the results of several studies on MRM in high-risk patient samples, the study gave arguments against a commonly spread presumption that a lack of specificity should be a reason against the use of MRM beyond common indications [7, 8, 17].

Another argument mentioned against a possible role of MRM in a broader set of indications has always been its cost-effectiveness compared to conventional imaging, which—as of today—is yet to be verified.

The determination of the cost-effectiveness of MRM in women at intermediate risk due to dense breasts so far was difficult, as data on the accuracy of MRM was mainly available in high-risk patient collectives.

Additionally, data suggested that reader experience may have a considerable impact on accuracy and therefore on the cost-effectiveness of MRM. Experience levels in MRM are still reported to be heterogeneous, also as a result of limited utilization of MRM [9].

Therefore, concerns regarding the economic feasibility of an extension of the current set of indications for MRM remain popular [18].

The aim of this study is to assess the economic impact of MRM, based on the data from our previously published, prospective TK-Study collective, examining patients with dense breasts in a stand-alone setting outside the current list of indications.

## Material and methods

### Patient collective

For this cost-effectiveness analysis, the results for the accuracy of MRM as a solitary imaging tool in women at intermediate risk due to dense breasts as part of the prospective TK-Study [9] were examined. Between April 2006 and December 2011, a consecutive total of 1488 women were prospectively examined. Of 1488 included patients, 393 patients were lost to follow-up, and 1095 patients were evaluated. One hundred twenty-four patients were diagnosed with malignancy by DCE-MRI (76 true positive (TP), 48 false positive (FP), 971 true negative (TN), and 0 false negative (FN) cases). Positive cases were confirmed by histology and negative cases by MR follow-ups or patient questionnaires over the next 5 years in 1737 cases (sensitivity 100%; specificity 95.2%; positive predictive value (PPV) 61.3%; negative predictive value (NPV) 100%; accuracy 95.5%). For invasive cancers only (DCIS excluded), the results were 63 TP, 27 FP, 971 TP, and 0 FN cases (sensitivity 100%; specificity 97.2%; PPV 70%; NPV 100%; accuracy 97.5%) [9].

### Model overview

**Fig. 1:**
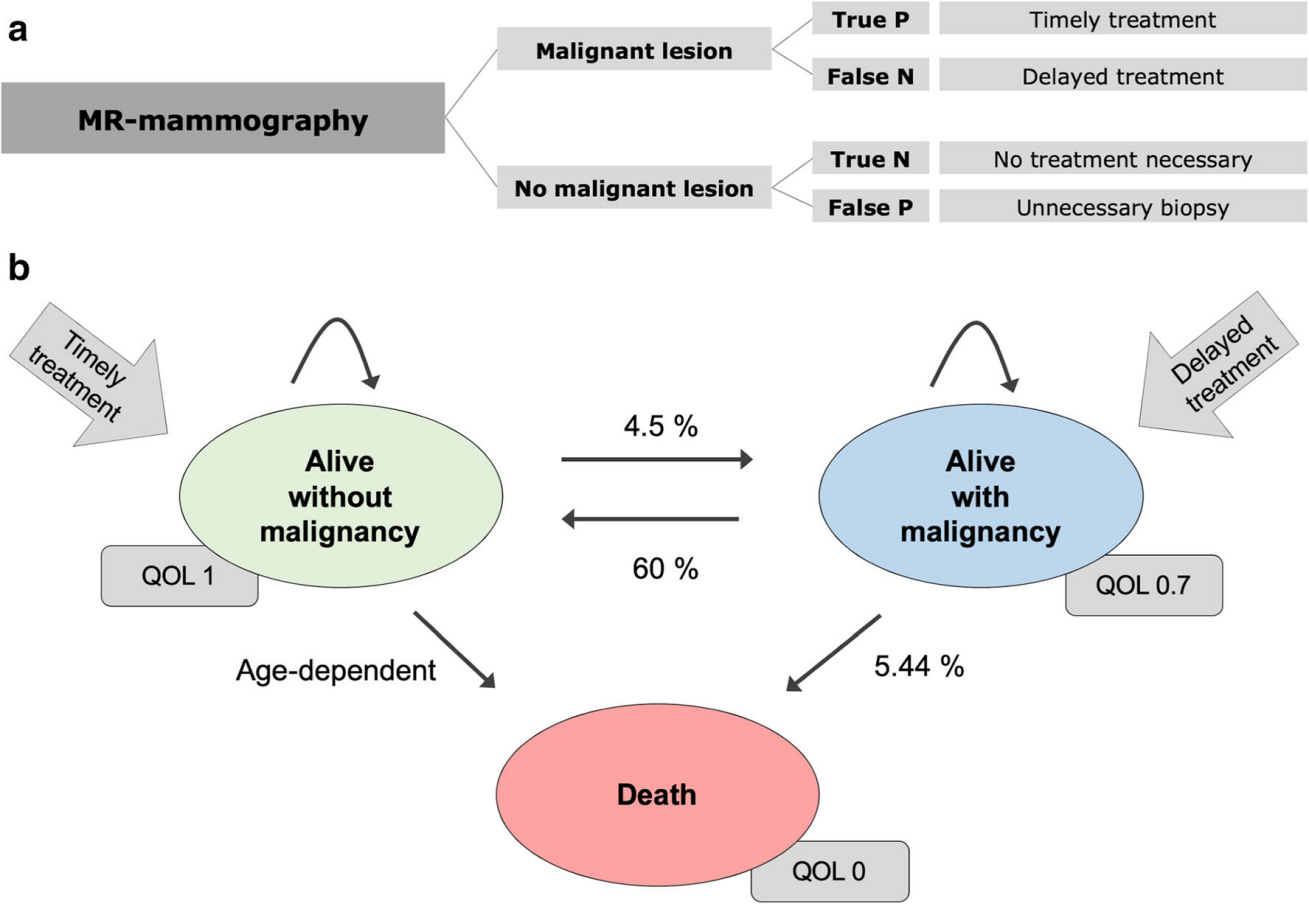
**a** Tree structure for MR-mammography strategy. For every potential outcome, respective Markov Models were applied. **b** Markov model estimating outcomes

For the economic evaluation, a Markov model discriminating between patients with and without malignancy (ground truth) was constructed (Fig. 1a). A Markov model represents certain relations between health states and corresponding costs and outcomes. In this context, the probability of true positive, false negative, true negative, and false positive results is delivered by the sensitivity and specificity of the underlying diagnostic tool—in our case MRM.

Depending on the diagnostic results, the corresponding state in a Markov chain model was assigned. These Markov states were “Alive - No cancer present,” “Alive - Present cancer,” and “Dead” (Fig. 1b). Corresponding costs in € and quality of life estimated in quality-adjusted life years (QALYs) were assigned to each state. The cumulative outcomes and costs were calculated as the sum over a 5-year period.

### Input parameters

Input parameters for the analysis were derived from published and unpublished data collected in the TK-Study. For the Markov model, input parameters were derived from the published literature. The model input parameters are summarized in Table 1.

**Table 1:** Model input parameters

Name	Estimate	Distribution	Source
Pre-test probability of malignancy	7.00%	*β*	Kaiser et al (2015) [9]
Average age at enrollment in the TK-Study	32 years	*β*	Kaiser et al (2015) [9]
Assumed WTP	100,000 €/QALY	*β*	Sanders et al (2016) [19]
Discount rate	3.00%	*β*	Sanders et al (2016) [19]
Markov model time	5 years	*β*	TK-Study
Diagnostic test performances			
Biopsy sensitivity	100%	*β*	Assumption
Biopsy specificity	100%	*β*	Assumption
MRM sensitivity	100%	*β*	Kaiser et al (2015) [9]
MRM specificity	97%	*β*	Kaiser et al (2015) [9]
Costs (acute)			
MRM	418.50 €	*γ*	TK-Study
Biopsy	300.00 €	*γ*	TK-Study
Early surgery	4000.00 €	*γ*	TK-Study
Delayed surgery	10,000.00 €	*γ*	TK-Study
Costs (long term)			
Yearly costs without tumor	0.00 €	*γ*	Assumption
Yearly costs with tumor	10,000.00 €	*γ*	Gruber et al (2012) [20]
Utilities			
QOL of patients without tumor	1	*β*	Assumption
QOL of patients with tumor	0.7	*β*	Sharma and Purkayastha (2017) [21]
Lost QALYs for biopsy	0.05	*β*	Assumption
Death	0	*β*	Assumption
Transition probabilities			
Probability of death without tumor per year	Age-dependent	*β*	Arias et al (2017) [22]
Probability of death with tumor per year	5.44%	*β*	Howlader et al (2019) [23]
Probability of recurrence per year	4.50%	*β*	Colleoni et al (2016) [24]
Probability of successful treatment per year	60.00%	*β*	Assumption

*MRM* MR-mammography, *QOL* quality of life, *WTP* willingness to pay

#### Diagnostic efficacy parameters

The diagnostic efficacy was adopted from the published data of TK-Study (see above). For each positive diagnostic finding, a consecutive biopsy was assumed. For modeling purposes, sensitivity and specificity of a biopsy were set to 100%, assuming it revealed the ground truth.

#### Utilities

For the assessment of the quality of life (QOL), respective utilities were derived from the literature. The QOL of patients without cancer was assumed to be not impaired, whereas the QOL of patients with present tumors was assumed to be reduced. Due to the relatively early tumor stage of the patients in the TK-Study at the time of diagnosis [7, 8, 25], 0.7 QALY was assumed to be the QOL [21].

#### Cost estimates

The direct costs of MRM were negotiated with a participating national insurance provider to the amount of 418.50 €. Costs for biopsy and surgery variations were drawn from the local reimbursement database of our university hospital and resulted in biopsy costs of approximately 300.00 € and 4000.00 € and 10,000.00 €, respectively, for an early vs. delayed surgery. These estimates were based on the assumption that the majority of biopsies are performed as ultrasound-guided or stereotactically XM-guided biopsies. A delayed diagnosis was assumed to be associated with more *extensive* surgery in higher-stage tumors.

Furthermore, long-term costs in patients with persisting cancer were set to 10,000.00 € per year, based on a conservative estimate [20]. Given a relatively young patient age in the study collective, the breast cancer–attributable costs in this age group may be considered particularly high.

#### Transition probabilities

Transition probabilities for the Markov model were derived from the published literature (Table 1). The risk of death without present cancer was derived from the US life tables [22]. According to the TK-Study, the prevalence of breast cancer was assumed to be 7.00% [9]. Further transition probabilities were derived from the published literature [26].

### Economic analysis

The economic analysis was performed in a dedicated decision analysis software program (TreeAge Pro Healthcare 2020, version 20.1.0, TreeAge Software Inc.).

#### Cost and cost-effectiveness analysis

For a base case scenario, the total costs during the 5-year simulation period were estimated along with the cumulative QOL in QALYs. Costs and outcomes were discounted with a yearly rate of 3.0%. Also, a willingness to pay (WTP) threshold of up to 100,000 € per QALY was regarded as acceptable in this setting as described in the literature [27].

#### Sensitivity analysis

To evaluate the stability of the model with respect to its input parameters, deterministic sensitivity analyses, estimating the impact of input parameters on total costs and cumulative effectiveness in the study period, were performed. Given that diagnostic efficacy measures and pre-test probability have a significant impact on expected costs and outcomes, the sensitivity and specificity of MRM as well as the probability of the presence of a malignant lesion were incorporated into the analysis. Furthermore, for the estimation of costs, the cost of MRM itself was investigated regarding its influence on overall costs.

## Results

### Outcome modeling

**Fig. 2:**
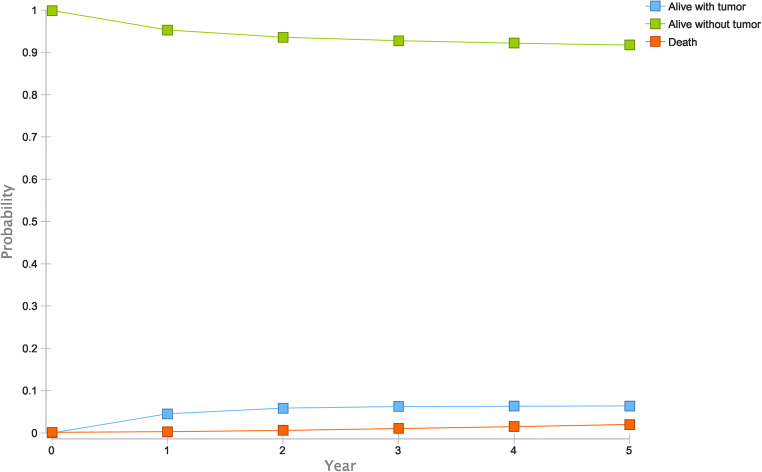
Markov modeling for timely diagnosis. Estimated outcomes for patients after timely diagnosis and after first treatment approach treatment for the 5-year period

Outcomes of patients were simulated in the Markov model defined above for a 5-year period to account for outcome differences of timely and delayed diagnosis. For patients with an initial true positive diagnosis, the status “Alive without malignancy” was assigned as a starting point, assuming immediate successful treatment after diagnosis. True negative and false positive patients were assigned “Alive without malignancy.” The simulated outcomes for these patients are summarized in Fig. 2.

A timely diagnosis (true positive) in patients resulted not only in significantly lower overall costs due to reduced treatment costs but also in an expected gain of 0.67 QALY when compared to a delayed diagnosis due to false negative results. While the expected cost for a true positive finding in the period was 7606.80 € with corresponding 4.62 QALYs, a false negative finding resulted in expected costs of 17,518.54 € with 3.95 QALYs. The corresponding costs and cumulative outcomes per patient are summarized in Table 2.

**Table 2:** Modeled results based on the decision model and Markov model

Status	Expected costs	Expected outcomes
True positive	7606.80 €	4.62 QALYs
False negative	17,518.54 €	3.95 QALYs
True negative	1193.16 €	4.70 QALYs
False positive	1493.16 €	4.65 QALYs

Calculated per-patient costs and outcomes for each scenario

### Cost-effectiveness analysis

Derived from the Markov model results, the overall model results were calculated: In the base case calculation of the model, MRM resulted in expected costs of 1650.48 € over a 5-year period with an expected cumulative outcome of 4.69 QALYs.

### Deterministic sensitivity analysis

#### Effect on total costs

**Fig. 3:**
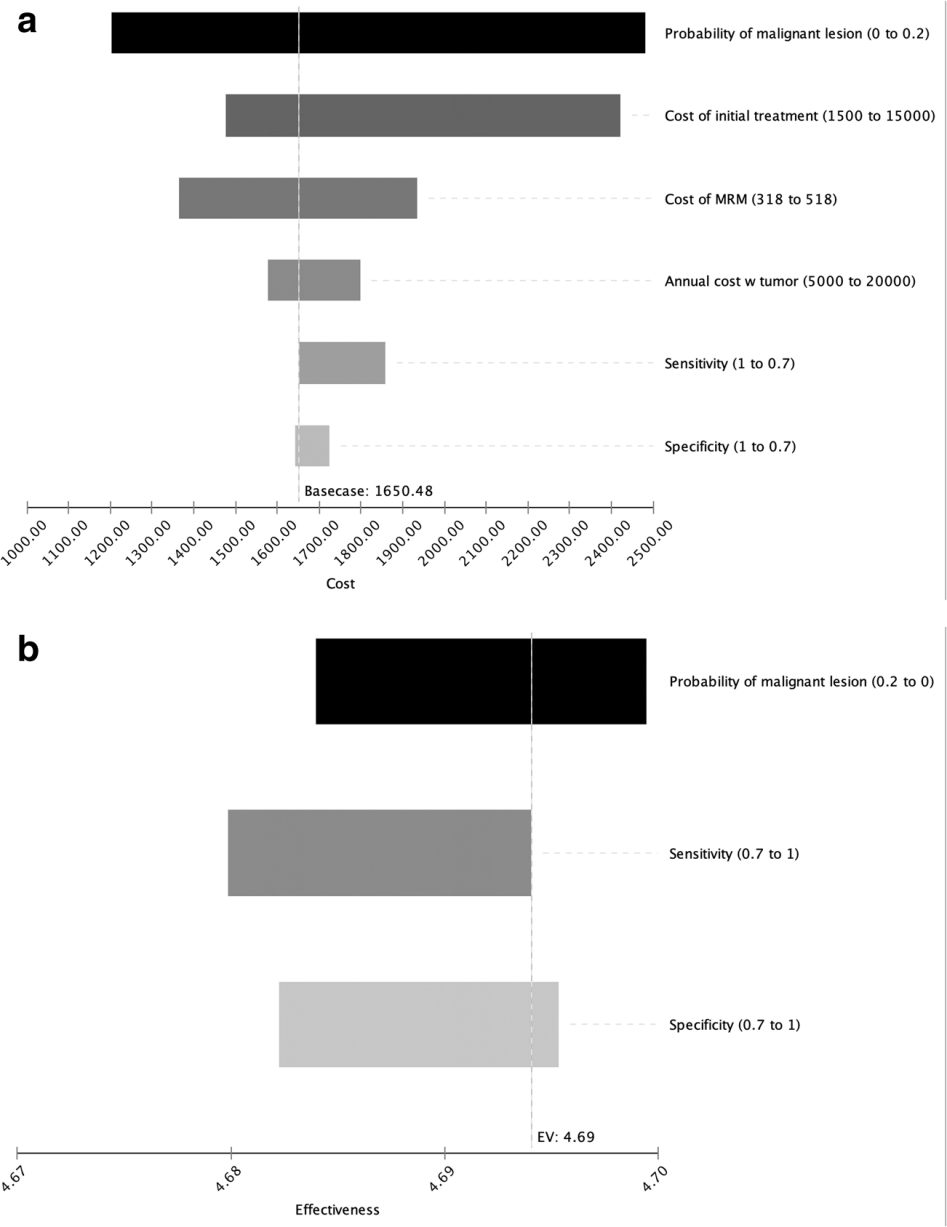
**a** Deterministic sensitivity analysis for costs—modeled expected costs (in €) of MR-mammography (MRM) strategy in the 5-year period. Pre-test probability and treatment costs are the most important driver of overall costs. **b** Deterministic sensitivity analysis for effectiveness—modeled expected outcomes of MRM strategy in the 5-year period. Decreased sensitivity and specificity affect quality of life significantly

To simulate the effect of input criteria on overall costs, a respective deterministic sensitivity analysis was performed. As the cost of treatment in patients with cancer had an important impact on the total costs, the pre-test probability of malignancy was identified as the most important driver when compared to the cost of MRM examinations, as well as differences in diagnostic performance (Fig. 2). However, the expected total costs in the 5-year period did not exceed 2500 € for the analyzed ranges (Fig. 3a).

#### Effect on cumulative outcomes

For the assessment of impact on overall effectiveness, a corresponding deterministic sensitivity analysis was performed (Fig. 3b). Based on varying probabilities of the presence of a malignant lesion (range from 0 to 20%) and varying sensitivity (70 to 100%) and specificity (70 to 100%) levels, the overall effectiveness ranged from 4.68 to 4.70 QALYs (Fig. 3b).

## Discussion

The TK-Study in its two parts, to our knowledge, was the first study to assess both the diagnostic accuracy and the economic significance of MRM in patients at intermediate risk of breast cancer due to their dense breast tissue.

In the first part of the study [9], the authors could find evidence for a high diagnostic accuracy of MRM in patients at intermediate risk due to dense breasts as a solitary imaging tool, i.e., without the help of conventional imaging, in line with quite recent and well-published data [28].

This scientific sequel about the economic significance illustrates that MRM may well be considered cost-effective in this novel patient cohort, suggesting an adaption of international guidelines, currently indicating MRM in high-risk patients only, along with a small role as problem solver [12, 13, 29] in special cases.

The results of this publication indicate clinical impact, as they suggest utilizing MRM in patients with dense breasts, if necessary, as a solitary imaging technique. MRM may be the method of choice not only in terms of accuracy in the detection of small tumors [30, 31] but also in terms of cost-effectiveness in patients with dense breasts, i.e., in patients, where conventional imaging is facing problems in accuracy due to breast density.

As this economic analysis is based on the prospective data of the TK-Study, the results have to be interpreted in the context of its input parameters: Due to its non-comparative nature, this study investigated the overall costs of MRM, uncompared to any other diagnostic modality, claiming cost-effectiveness only within the range of the WTP.

In other words, within the limits of the price range, most international healthcare systems are willing to pay for an additional QALY, and MRM in dense breasts in a setting unaccompanied by conventional imaging may be considered well within the affordable range of the broad spectrum of the WTP. This is supported by the results for false negative patients in the study: Patients receiving a false negative diagnosis are associated with high upstream costs in therapy as well as lowered quality of life, preventable by the high sensitivity of MRM.

In terms of diagnostic accuracy, most recent and high-ranking published data [28, 32] comparing conventional breast imaging and MRM is in line with our results. Thus, accuracy as well as its economic interpretation in this publication may be considered consistent.

Literature describes economic aspects of XM and DBT in the context of screening programs [33, 34]. First results on the economic implications of MRM have also been published [35]: Ahern et al [27] investigated the cost-effectiveness of MRM in the context of high-risk patients reporting ICER values consistently below the WTP threshold of $100,000/QALY, supporting our results. Ahern et al [27] did apply conservative results for MRM for their cost-effectiveness analysis, assuming a relatively low specificity for MRM, yet still confirming its cost-effectiveness.

Our analysis provides novel impact as it was conducted within a completely new patient indication (intermediate risk due to dense breasts) and was based on prospectively generated data.

Literature on the cost-effectiveness of MRM is based on its majority on conservative results of diagnostic performance and may need re-evaluation in the light of recently published literature.

The modeling performed in this study has to be interpreted in the context of its input parameters: First, the underlying hypothesis in this patient collective is that the diagnosis is achieved in early stages of the disease (Tis or T1) in the vast majority of cases. This assumption is in line with the published literature [7, 8, 28] and leads to the further premise that a M1 stage should be very uncommon in these patients. These results are reflected in the Markov modeling presented above.

From the sensitivity analyses, certain conclusions can be drawn: First, the pre-test probability of malignancy has the most notable impact on overall costs. This is due to the fact that the impact of potentially necessary therapy on costs (opportunity costs) is vastly outnumbering the impact of diagnostic costs—even assuming *costly* examinations of MRM. Furthermore, a loss in sensitivity and specificity is associated with a significant increase in overall costs (Fig. 3a) due to an increased number of false positive and false negative patients. Therefore, a head-to-head comparison with other modalities would be of high relevance in future investigations. Likewise, a loss in diagnostic accuracy was associated with quality-of-life losses. The results point at the fact that an additional investment in sensitive diagnostic modalities such as MRM may be well justified, as they result in better outcomes and lower resulting therapeutic costs. However, the results of our analysis also show a certain importance of a high level of experience of MRM readers, given that a lower accuracy is associated with losses of quality of life (Fig. 3b). This is important, given that a prerequisite for the high accuracy of MRM in the TK-Study was reader experience.

In conclusion, our results suggest MRM to be a cost-effective as well as accurate diagnostic option in patients at intermediate risk of breast cancer due to dense breasts. Further studies examining the cost-effectiveness of MRM in women of average risk should be a matter of future research, also investigating the cost-effectiveness of other imaging techniques.

## Abbreviations

MRM: MR-mammography
QALY: Quality-adjusted life year
WTP: Willingness to pay
XM: X-ray-based mammography

## References

[1] Pisano ED, Hendrick RE, Yaffe MJ (2008). Diagnostic accuracy of digital versus film mammography: exploratory analysis of selected population subgroups in DMIST. Radiology.

[2] Kalager M (2014). Overdiagnosis in breast cancer screening: women have minimal prior awareness of the issue, and their screening intentions are influenced by the size of the risk. Evid Based Nurs.

[3] Paci E, EUROSCREEN Working Group (2012). Summary of the evidence of breast cancer service screening outcomes in Europe and first estimate of the benefit and harm balance sheet. J Med Screen.

[4] Mittmann N, Stout NK, Lee P (2015). Total cost-effectiveness of mammography screening strategies. Health Rep.

[5] Pataky R, Ismail Z, Coldman AJ (2014). Cost-effectiveness of annual versus biennial screening mammography for women with high mammographic breast density. J Med Screen.

[6] Tina Shih Y-C, Dong W, Xu Y, Shen Y (2019). Assessing the cost-effectiveness of updated breast cancer screening guidelines for average-risk women. Value Health.

[7] Kuhl C, Weigel S, Schrading S et al (2010) Prospective multicenter cohort study to refine management recommendations for women at elevated familial risk of breast cancer: the EVA trial. J Clin Oncol 28:1450–1457. 10.1200/JCO.2009.23.0839 10.1200/JCO.2009.23.0839 20177029

[8] Sardanelli F, Podo F, Santoro F (2011). Multicenter surveillance of women at high genetic breast cancer risk using mammography, ultrasonography, and contrast-enhanced magnetic resonance imaging (the high breast cancer risk Italian 1 study): final results. Invest Radiol.

[9] Kaiser CG, Reich C, Dietzel M (2015). DCE-MRI of the breast in a stand-alone setting outside a complementary strategy - results of the TK-study. Eur Radiol.

[10] Bennani-Baiti B, Bennani-Baiti N, Baltzer PA (2016). Diagnostic performance of breast magnetic resonance imaging in non-calcified equivocal breast findings: results from a systematic review and meta-analysis. PLoS One.

[11] Bennani-Baiti B, Baltzer PA (2017). MR imaging for diagnosis of malignancy in mammographic microcalcifications: a systematic review and meta-analysis. Radiology.

[12] Sardanelli F, Boetes C, Borisch B (2010). Magnetic resonance imaging of the breast: recommendations from the EUSOMA working group. Eur J Cancer.

[13] Sardanelli F, Aase HS, Álvarez M (2017). Position paper on screening for breast cancer by the European Society of Breast Imaging (EUSOBI) and 30 national breast radiology bodies from Austria, Belgium, Bosnia and Herzegovina, Bulgaria, Croatia, Czech Republic, Denmark, Estonia, Finland, France, Germany, Greece, Hungary, Iceland, Ireland, Italy, Israel, Lithuania, Moldova, The Netherlands, Norway, Poland, Portugal, Romania, Serbia, Slovakia, Spain, Sweden, Switzerland and Turkey. Eur Radiol.

[14] Kriege M, Brekelmans CTM, Boetes C (2004). Efficacy of MRI and mammography for breast-cancer screening in women with a familial or genetic predisposition. N Engl J Med.

[15] Strobel K, Schrading S, Hansen NL, Barabasch A, Kuhl CK (2014) Assessment of BI-RADS category 4 lesions detected with screening mammography and screening US: utility of MR imaging. Radiology 140645. 10.1148/radiol.14140645 10.1148/radiol.14140645 25271857

[16] Monticciolo DL, Newell MS, Moy L, Niell B, Monsees B, Sickles EA (2018) Breast cancer screening in women at higher-than-average risk: recommendations from the ACR. J Am Coll Radiol 15:408–414. 10.1016/j.jacr.2017.11.034 10.1016/j.jacr.2017.11.034 29371086

[17] Boetes C, Mus RD, Holland R (1995). Breast tumors: comparative accuracy of MR imaging relative to mammography and US for demonstrating extent. Radiology.

[18] Mann RM, Kuhl CK, Moy L (2019). Contrast-enhanced MRI for breast cancer screening. J Magn Reson Imaging.

[19] Sanders GD, Neumann PJ, Basu A et al (2016) Recommendations for conduct, methodological practices, and reporting of cost-effectiveness analyses. JAMA 316:1093. 10.1001/jama.2016.12195 10.1001/jama.2016.12195 27623463

[20] Gruber EV, Stock S, Stollenwerk B (2012). Breast cancer attributable costs in Germany: a top-down approach based on sickness funds data. PLoS One.

[21] Sharma N, Purkayastha A (2017). Factors affecting quality of life in breast cancer patients: a descriptive and cross-sectional study with review of literature. J Life Health.

[22] Arias E, Heron M, Xu J (2017). United States life tables, 2013. Natl Vital Stat Rep.

[23] Howlader N, Noone AM, Krapcho M, Miller D, Brest A, Yu M (2019) SEER cancer statistics review, 1975–2016. Bethesda, MD: National Cancer Institute 1423–37

[24] Colleoni M, Sun Z, Price KN et al (2016) Annual hazard rates of recurrence for breast cancer during 24 years of follow-up: results from the international breast cancer study group trials I to V. J Clin Oncol 34:927–935 10.1200/JCO.2015.62.3504 PMC4933127 26786933

[25] Saadatmand S, Geuzinge HA, Rutgers EJT (2019). MRI versus mammography for breast cancer screening in women with familial risk (FaMRIsc): a multicentre, randomised, controlled trial. Lancet Oncol.

[26] Cancer of the breast (female) - cancer stat facts. In: SEER. https://seer.cancer.gov/statfacts/html/breast.html. Accessed 3 Mar 2020

[27] Ahern CH, Shih Y-CT, Dong W, Parmigiani G, Shen Y (2014) Cost-effectiveness of alternative strategies for integrating MRI into breast cancer screening for women at high risk. Br J Cancer 111:1542–1551. 10.1038/bjc.2014.458 10.1038/bjc.2014.458 PMC4200098 25137022

[28] Bakker MF, de Lange SV, Pijnappel RM (2019). Supplemental MRI screening for women with extremely dense breast tissue. N Engl J Med.

[29] Mann RM, Kuhl CK, Kinkel K, Boetes C (2008). Breast MRI: guidelines from the European Society of Breast Imaging. Eur Radiol.

[30] Lehman CD, Blume JD, Thickman D (2005). Added cancer yield of MRI in screening the contralateral breast of women recently diagnosed with breast cancer: results from the International Breast Magnetic Resonance Consortium (IBMC) trial. J Surg Oncol.

[31] Tilanus-Linthorst MM, Obdeijn IM, Bartels KC, de Koning HJ, Oudkerk M (2000) First experiences in screening women at high risk for breast cancer with MR imaging. Breast Cancer Res Treat 63:53–60 10.1023/a:1006480106487 11079159

[32] Comstock CE, Gatsonis C, Newstead GM (2020). Comparison of abbreviated breast MRI vs digital breast tomosynthesis for breast cancer detection among women with dense breasts undergoing screening. JAMA.

[33] Allaire BT, Ekweme D, Hoerger TJ (2019). Cost-effectiveness of patient navigation for breast cancer screening in the National Breast and Cervical Cancer Early Detection Program. Cancer Causes Control.

[34] Lowry KP, Trentham-Dietz A, Schechter CB et al (2019) Long-term outcomes and cost-effectiveness of breast cancer screening with digital breast tomosynthesis in the United States. J Natl Cancer Inst. 10.1093/jnci/djz184 10.1093/jnci/djz184 PMC7301096 31503283

[35] Kaiser CG, Reich C, Wasser K, Schönberg SO, Kaiser WA (2012) Economic aspects of MR-mammography in dense breasts. Eur J Radiol 81(Suppl 1):S69–S71. 10.1016/S0720-048X(12)70027-X 10.1016/S0720-048X(12)70027-X 23083609

